# Propofol inhibits the release of interleukin-6, 8 and tumor necrosis factor-α correlating with high-mobility group box 1 expression in lipopolysaccharides-stimulated RAW 264.7 cells

**DOI:** 10.1186/s12871-017-0441-0

**Published:** 2017-10-26

**Authors:** Jie Jia, Yijuan Sun, Zurong Hu, Yi Li, Xiangcai Ruan

**Affiliations:** 1 Department of Anesthesiology, The First Affiliated Hospital of Jinan University, Guangzhou, China; 2 Department of Anesthesiology, Guangdong Women and Children Hospital, Guangzhou, China; 3 Department of Gynecology, Guangdong Women and Children Hospital, Guangzhou, China; 4 Department of Anesthesiology, Guangzhou First People’s Hospital, Affiliated Hospital of Guangzhou Medical University, No. 1 Panfu Road, Guangzhou, 510180 China

**Keywords:** Lipopolysaccharide, Propofol, High-mobility group box 1, Interleukin-6, Interleukin-8, Tumor necrosis factor-α

## Abstract

**Background:**

Studies have found that propofol can inhibit endotoxin-induced monocyte-macrophages to produce various inflammatory factors. This study is to disclose whether the propofol affects the expression of high-mobility group box 1 (HMGB1) in lipopolysaccharides (LPS)-stimulated RAW 264.7 cells and the release of interleukin-6 (IL-6), 8 (IL-8) and tumor necrosis factor-α (TNF-α).

**Methods:**

RAW 264.7 cells were divided into four groups for intervention. After culturing for 16 h, the cells and culture supernatants were collected. The expression of HMGB1 in RAW 264.7 cells was detected by Western blot. The levels of IL-6, IL-8 and TNF-α in supernatants of cells were determined by enzyme-linked immunosorbent assay (ELISA).

**Results:**

Stimulation of LPS increased the expression of HMGB1 and promoted the release of IL-6, IL-8 and TNF-α in supernatants of RAW 264.7 cells (*p* < 0.05); however, propofol down-regulated the expression of LPS-stimulated HMGB1 and reduced the LPS-stimulated releases of IL-6, IL-8 and TNF-α in supernatants of RAW 264.7 cells (*p* < 0.05). Moreover, the releases of IL-6, IL-8 and TNF-α intimately correlated with the expression of HMGB1 in this process (*p* < 0.05).

**Conclusion:**

Propofol inhibited the releases of IL-6, IL-8 and TNF-α in LPS-stimulated RAW 264.7 cells, and the levels of IL-6, IL-8 and TNF-α intimately correlated with the expression of HMGB1, which indicating that propofol may prevent inflammatory responses through reducing the releases of these cytokines and inflammatory mediators.

## Background

Propofol is used for induction and maintenance of general anesthesia. It is often used with epidural or spinal anesthesia, and is often used with analgesics, muscle relaxants and inhalation anesthetics. Previous studies have used the model of ischemia-reperfusion injury to explore the inflammatory process and found that propofol exhibited antioxidant and anti-inflammatory effects [1–5]. Lipopolysaccharide (LPS) is the major cause of clinical fever, it is also the key factor that cause the shock, sepsis, cholera, and other diseases like adult respiratory syndrome, multiple organ failure, or the pathological processes [6]. Gram-negative bacteria are often used to induce inflammation in vitro, and studies have shown that LPS is a major component of the outer membrane of Gram-negative bacteria [6]. Studies have confirmed that high-mobility group box 1 (HMGB1) is a DNA-binding protein that maintains nucleosomal structures and regulates gene transcription, which is characterized by highly conserved proteins. Recent studies have suggested that it exhibits a pronounced proinflammatory effect in the inflammatory response [7], which is involved in the process of development of sepsis, as well as the important inflammatory mediators for the late period in the lethal effects of LPS [8]. HMGB1 appears to activate macrophages leading to the secretion of multiple cytokines, which in turn cause extensive inflammatory responses, including intercellular adhesion molecule 1 (ICAM-1), vascular cell adhesion molecule 1 (VCAM-1), tumor necrosis factor-α (TNF-α), interleukin-1β (IL-1β) and nitric oxide [9].

Studies have found that propofol can block endotoxin-induced monocyte-macrophages to produce various inflammatory factors [3, 5, 9]. LPS stimulation can induce HMGB1 release from mouse macrophages, however, propofol can inhibit this process leading to downregulation of HMGB1 mRNA and also block the activation of nuclear transcription factor-κB (NF-κB) [10]. In addition, the propofol can inhibit the expression of toll-like receptor 4 (TLR-4) and NF-κB to block the activation of p38 mitogen-activated protein kinase and the expression of pro-inflammatory cytokines [4]. However, the actions of propofol to regulate the expressions of interleukin-6 (IL-6), 8 (IL-8) and TNF-α and the correlation with HMGB1 in LPS-stimulated murine macrophage are unclear. Based on the importance of HMGB1 in immunoreactions and the potential relationship between HMGB1 and LPS, the aim of our study is to disclose whether LPS stimulation can lead to RAW 264.7 cells (murine macrophage) secreting more HMGB1, IL-6, IL-8 and TNF-α. However, the addition of propofol can down-regulate the expression of LPS-stimulated HMGB1, IL-6, IL-8 and TNF-α. And the levels of IL-6, IL-8 and TNF-α seems to correlate with the expression of HMGB1 in this process.

## Methods

### Preparation and culture of cells

Mouse monocyte/macrophage leukemia RAW264.7 cells were purchased from Shanghai Cell Biology cell bank. RPMI-1640 and fetal bovine serum (fetal bovine serum, FBS) were purchased from Gibco BRL Company. LPS was purchased from the United States (*Escherichia coli* O11: B4) Sigma Corporation. The isolated RAW264.7 cells were collected into the centrifuge tube. The cells were centrifuged for 10 min at 1000 r/min. The supernatants was then discarded and the cells were pooled with RPMI 1640 medium containing 20% FBS with 1% sodium pyruvate and 1% streptomycin. After the cell density was adjusted, the cells were cultured at 37 °C under 5% CO2. The culture medium was replaced by 2–3 days according to conditional of cell growth.

### Grouping of cell experiments

Three days prior to the experiment, the cells were seeded into a six-well plate and replaced with serum-free medium 6 h prior to the experiment and then grouped. The experimental cells were divided into four groups, which are showed as follows: (1) Blank control group (without any intervention); (2) LPS intervention group, plus 250 ng/mL LPS; (3) Low-dose group of propofol treatment, plus 250 ng/mL LPS and 25 μmol/L propofol; (4) High-dose group of propofol treatment, plus 250 ng/mL LPS and 50 μmol/L propofol. Each group of cells was cultured at 37 °C under 5% CO2 and cells were collected after 16 h culturing (the same culture conditions).

### Western blotting

The cells in the logarithmic growth phase were harvested and washed once with phosphate phosphate saline (PBS), and the total protein was extracted according to the method described in the protein extraction kit. The extracted protein was boiled for 5 min with 2× sodium dodecyl sulfate (SDS) -polyacrylamide gel electrophoresis (PAGE) sample buffer and 20% glycerol. Each protein sample was then assayed for concentration with Micro-BCA protein. The protein samples were gel electrophected with 12% SDS-PAGE and 30 μg of total cell protein was added to each lane. Proteins were transferred to the polyvinylidene difluoride membrane (PVDF membrane) by electrophoresis. The PVDF membrane was then blocked at 4 °C with TBST buffer (pH 7.4, TBS added 0.1% Tween-20) containing 5% skimmed milk powder. PVDF membranes were incubated with anti-HMGB1 antibody (1: 500 dilution) (product code: BM3965; Boster Biological Technology co.ltd, Wuhan, China) for 1 h at 37 °C. We used β-actin as the internal control. PVDF membranes were rinsed three times with TBST buffer and incubated with HRP-conjugated secondary antibody (1: 1000) at room temperature for 1 h. Positive signals were visualized using the enhanced chemiluminescence method. Quantitative expression of protein and expression images were detected and collected through the Kodak IS2000R multifunctional image workstation.

### Enzyme-linked immunosorbent assay (ELISA)

According to the previous grouping method, the RAW264.7 cell suspension was added to the 6-well plate, 1 mL per well, and 6 wells in each group. The cells were incubated at 37 °C under 5% CO2. After 16 h of culturing, the supernatants were collected for further experiments. The levels of IL-6 (product code: 70-EK2062/2, Lianke Biotech Co., Ltd. Shenzhen, China), IL-8 (product code: ZK-M4810, Ziker Biotech Co., Ltd. Shenzhen, China) and TNF-α (product code: B-21672, Dice Biotech Co., Ltd. Hangzhou, China) in the supernatants of cells were measured by ELISA according to the experimental method provided by the reagent manufacturer. The concentrations of IL-6, IL-8 and TNF-α were calculated based on standard curves provided with the kits, and the results of TNF-α and IL-8 were expressed in ng/mL, and IL-6 in pg/mL.

### Statistical analysis

The mean ± standard deviation (M ± SD) is calculated for the continuity variable data and the relevant statistical analysis is performed. The comparison for different expression levels of HMGB1, TNF-α, IL-6 and IL-8 in different experimental groups was calculated by the Student’s *T*-test (matched samples), One-WAY ANOVA (single factor analysis) and non-parametric rank correlation analysis according to different data characteristics. The statistical analysis was finished with SPSS 19.0 (SPSS, Chicago, USA) software. All tests of statistical significance were two-sided, and a *p*-value <0.05 was considered as statistically significant.

## Results

### LPS stimulation increased the expression of HMGB1 in RAW 264.7 cells

**Fig. 1:**
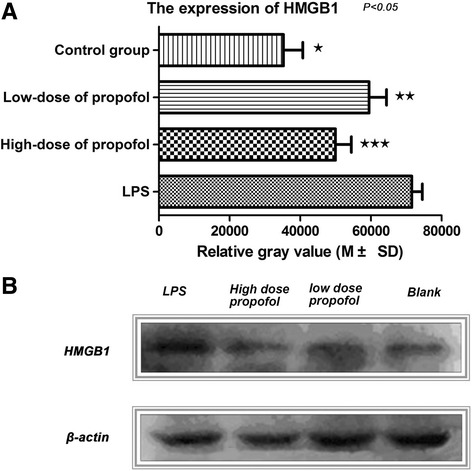
The expression of HMGB1 between different intervention groups by western blotting in RAW 264.7 cells. **a** Semi-quantitative analysis of western blotting showed that the expression of HMGB1 in blank control group ^★^ was lower than LPS intervention group ^★★^, low-dose group of propofol and high-dose group of propofol ^★★★^ respectively (*p* < 0.05); and, the expression of HMGB1 in low-dose group of propofol ^★★^ and high-dose group of propofol ^★★★^ was all lower than LPS stimulation group (*p* < 0.05); **b** The results of western blotting showed that the expression of HMGB1 in intervention groups stimulated by LPS was up-regulated, compared with the blank control group; however, the expression of HMGB1 in low-dose group of propofol and high-dose group of propofol was all lower than LPS stimulation group; Blank control group, without any intervention; LPS intervention group, plus 250 ng/mL LPS; Low-dose group of propofol treatment, plus 250 ng/mL LPS and 25 μmol/L propofol; High-dose group of propofol treatment, plus 250 ng / mL LPS and 50 μmol/L propofol LPS; Blank, control group; LPS, lipopolysaccharide; HMGB1, high-mobility group box 1

As shown in Table 1, after RAW 264.7 cells were stimulated by LPS for 16 h, the expression of HMGB1 protein in cells of LPS group was up-regulated compared with the blank control group (no addition of LPS and propofol). The test of western blotting showed that the absorbance values of HMGB1 protein was 36,010 ± 2550 in blank control group and 71,070 ± 2178 in LPS intervention group, suggesting that the expression of HMGB1 in LPS intervention group was higher than that in blank control group (*p* = 0.001). Compared with the blank control group (36,010 ± 2550), the expressions of HMGB1 in low-dose group of propofol (59,970 ± 2453) and the high-dose group (52,470 ± 2018) were also increased (*p* = 0.002, 0.012 respectively), showing that the expression of HMGB1 in all intervention groups was up-regulated, compared with the blank control group (Fig. 1a and b), which suggested that LPS stimulation increased the expression of HMGB1 in RAW 264.7 cells (*p* < 0.05).

**Table 1:** The relative gray value of HMGB1 protein band in different groups (*n* = 4)

Groups	M ± SD		Groups	M ± SD	Statistical value	*P* value
Blank control group	36,010 ± 2550	VS	LPS intervention	71,070 ± 2178	10.46	0.001
			Low-dose of propofol	59,970 ± 2453	6.772	0.002
			High-dose of propofol	52,470 ± 2018	4.313	0.012
LPS intervention	71,070 ± 2178		Low-dose of propofol	59,970 ± 2453	3.386	0.027
			High-dose of propofol	52,470 ± 2018	3.443	0.026
Low-dose of propofol	59,970 ± 2453		High-dose of propofol	52,470 ± 2018	5.955	0.004

*M ± SD* mean ± standard deviation, *Blank control group* without any intervention, *LPS intervention group* 250 ng/mL LPS, *Low-dose group of propofol treatment* plus 250 ng/mL LPS and 25 μmol/L propofol, *High-dose group of propofol treatment* plus 250 ng/mL LPS and 50 μmol/L propofol

### Addition of propofol down-regulated the expression of LPS-stimulated HMGB1

As shown in Table 1, compared with the LPS intervention group (71,070 ± 2178), the expressions of HMGB1 in propofol low dose group and high dose group were all significantly decreased, which showed 59,970 ± 2453 and 52,470 ± 2018 respectively (*p* = 0.027, 0.026 respectively) (Fig. 1a). In addition, the expression of HMGB1 in the propofol high dose group (59,970 ± 2453) was lower than that in the low dose group (52,470 ± 2018) (*p* = 0.004) (Table 1), which suggested that propofol down-regulated the expression of LPS-stimulated HMGB1 expression showing a concentration-dependent pattern. Similarly, the results of western blotting also showed that the expression of HMGB1 in different dose propofol groups was down-regulated, compared with the LPS intervention group (*p* < 0.05) (Fig. 1b).

### LPS stimulation increased the release of IL-6, IL-8 and TNF-α in RAW 264.7 cells

**Fig. 2:**
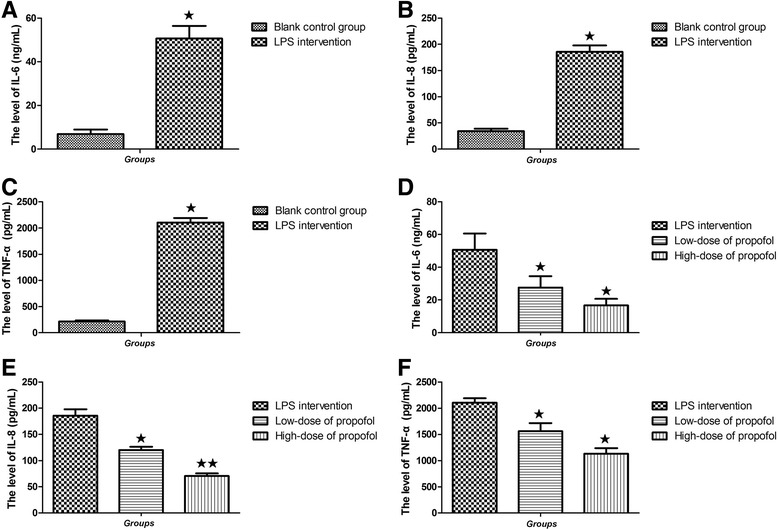
Propofol affected the release of IL-6, IL-8 and TNF-α by in LPS-stimulated RAW 264.7 cells. **a** The level of IL-6 in blank control group was lower than that in LPS intervention group (^★^ *p* < 0.05); **b** The level of IL-8 in blank control group was lower than that in LPS intervention group (^★^ *p* < 0.05). **c** The level of TNF-α in blank control group was lower than that in LPS intervention group (^★^ *p* < 0.05); **d**. Either in low-dose or in high-dose group of propofol, the level of IL-6 was all lower than that in LPS intervention group (^★^ *p* < 0.05); **e**. Either in low-dose or in high-dose group of propofol, the level of IL-8 was all lower than that in LPS intervention group (^★^ *p* < 0.05), especially, the level of IL-8 in the propofol high dose group was significantly lower than that in the low dose group (^★★^ *p* = 0.004); **f**. Either in low-dose or in high-dose group of propofol, the level of TNF-α was all lower than that in LPS intervention group (^★^ *p* < 0.05). Blank control group, without any intervention; LPS intervention group, plus 250 ng/mL LPS; Low-dose group of propofol treatment, plus 250 ng/mL LPS and 25 μmol/L propofol; High-dose group of propofol treatment, plus 250 ng/mL LPS and 50 μmol/L propofol; LPS, lipopolysaccharide; IL-6, interleukin- 6; IL-8, interleukin-8; TNF-α, tumor necrosis factor-α; HMGB1, high-mobility group box 1

After RAW 264.7 cells were stimulated by LPS for 16 h, we found that the value of IL-6 in LPS intervention group (50.64 ± 5.77 ng/mL) was higher than that in blank control group (6.89 ± 3.51 ng/mL) (*p* = 0.002) (Table 2, Fig. 2a). In addition, compared with the blank control group (34.54 ± 8.61 pg/mL), the expression of IL-8 in LPS intervention group (185.64 ± 21.12 pg/mL) was remarkably increased (*p* < 0.001) (Table 3, Fig. 2b). Moreover, the level of TNF-α in LPS intervention group (2102.34 ± 150.60 pg/mL) was also up-regulated, compared with the blank control group (213.20 ± 40.09 pg/mL) after LPS stimulation (*p* < 0.001) (Table 4, Fig. 2c). The results suggested that LPS stimulation increased the release of IL-6, IL-8 and TNF-α in RAW 264.7 cells.

**Table 2:** Effects of propofol on IL-6 secretion in RAW264.7 cells (M ± SD, *n* = 4)

Groups	M ± SD (ng/mL)		Groups	M ± SD (ng/mL)	Statistical value	*P* value
Blank control group	6.89 ± 3.51	VS	LPS intervention	50.64 ± 5.77	−7.150	0.002
			Low-dose of propofol	27.58 ± 4.04	−4.570	0.01
			High-dose of propofol	16.67 ± 4.30	−3.183	0.03
LPS intervention	50.64 ± 5.77		Low-dose of propofol	27.58 ± 4.04	3.272	0.031
			High-dose of propofol	16.67 ± 4.30	5.463	0.005
Low-dose of propofol	27.58 ± 4.04		High-dose of propofol	16.67 ± 4.30	2.344	0.079

*M ± SD* mean ± standard deviation, *Blank control group* without any intervention, *LPS intervention group* 250 ng/mL LPS, *Low-dose group of propofol treatment* plus 250 ng/mL LPS and 25 μmol/L propofol, *High-dose group of propofol treatment* plus 250 ng/mL LPS and 50 μmol/L propofol

**Table 3:** Effects of propofol on IL-8 secretion in RAW264.7 cells (M ± SD, *n* = 4)

Groups	M ± SD (pg/mL)		Groups	M ± SD (pg/mL)	Statistical value	*P* value
Blank control group	34.54 ± 8.61	VS	LPS intervention	185.64 ± 21.12	−11.646	0.0001
			Low-dose of propofol	120.03 ± 11.35	−10.887	0.0001
			High-dose of propofol	70.56 ± 9.19	−5.181	0.007
LPS intervention	185.64 ± 21.12		Low-dose of propofol	120.03 ± 11.35	4.794	0.009
			High-dose of propofol	70.56 ± 9.19	8.724	0.001
Low-dose of propofol	120.03 ± 11.35		High-dose of propofol	70.56 ± 9.19	6.029	0.004

*M ± SD* mean ± standard deviation, *Blank control group* without any intervention, *LPS intervention group* 250 ng/mL LPS, *Low-dose group of propofol treatment* plus 250 ng/mL LPS and 25 μmol/L propofol, *High-dose group of propofol treatment* plus 250 ng/mL LPS and 50 μmol/L propofol

**Table 4:** Effects of propofol on TNF-α secretion in RAW264.7 cells (M ± SD, *n* = 4)

Groups	M ± SD (pg/mL)		Groups	M ± SD (pg/mL)	Statistical value	*P* value
Blank control group	213.20 ± 40.09	VS	LPS intervention	2102.34 ± 150.60	−21.077	0.0001
			Low-dose of propofol	1562.20 ± 267.15	−8.656	0.001
			High-dose of propofol	1129.21 ± 187.96	−5.181	0.007
LPS intervention	2102.34 ± 150.60		Low-dose of propofol	1562.20 ± 267.15	3.055	0.038
			High-dose of propofol	1129.21 ± 187.96	7.031	0.002
Low-dose of propofol	1562.20 ± 267.15		High-dose of propofol	1129.21 ± 187.96	2.301	0.083

*M ± SD* mean ± standard deviation, *Blank control group* without any intervention, *LPS intervention group* 250 ng/mL LPS, *Low-dose group of propofol treatment* plus 250 ng/mL LPS and 25 μmol/L propofol, *High-dose group of propofol treatment* plus 250 ng/mL LPS and 50 μmol/L propofol

### Addition of propofol reduced the releases of IL-6, IL-8 and TNF-α in LPS stimulated-RAW 264.7 cells

However, the addition of propofol affects the release of LPS-stimulated IL-6, IL-8 and TNF-α in RAW 264.7 cells. Either in propofol low dose group (27.58 ± 4.04 ng/mL) or in high dose group (16.67 ± 4.30 ng/mL), we noticed that the levels of IL-6 were lower than that in LPS intervention group (50.64 ± 5.77 ng/mL) (*p* = 0.031, 0.005 respectively) (Table 2, Fig. 2d). In addition, the level of IL-8 in different dose propofol groups of propofol (120.03 ± 11.35 pg/mL for low dose; 70.56 ± 9.19 pg/mL for high dose) also decreased compared with LPS intervention group (185.64 ± 21.12 pg/mL) (*p* = 0.009, 0.001 respectively); especially, the level of IL-8 in the propofol high dose group (70.56 ± 9.19 pg/mL) was significantly lower than that in the low dose group (120.03 ± 11.35 pg/mL) (*p* = 0.004) (Table 3, Fig. 2e). Whether it is in propofol low-dose group (1562.20 ± 267.15 pg/mL) or in high-dose group (1129.21 ± 187.96 pg/mL), the level of TNF-α all displayed a significant decrease, compared with LPS intervention group (2102.34 ± 150.60 pg/mL) (*p* = 0.038, 0.002 respectively) (Table 4, Fig. 2f).

### The release of IL-6, IL-8 and TNF-α correlated with the expression of HMGB1 in RAW 264.7 cells interfered by LPS and propofol

**Fig. 3:**
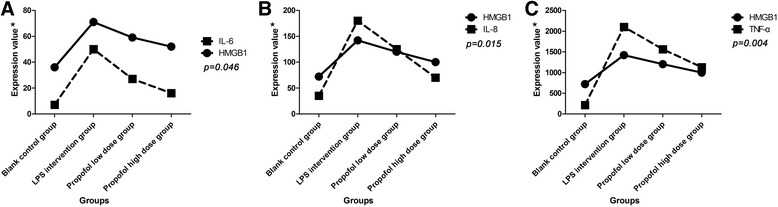
The relation between the release of IL-6, IL-8 and TNF-α and the expression of HMGB1 in supernatants of RAW 264.7 cells. **a** After propofol intervention in RAW 264.7 cells, the secretion of IL-6 and HMGB1 expression in RAW 264.7 cells had a close linear correlation (*p* < 0.05); with the decline in HMGB1 expression, IL-6 also showed a downward trend; **b** The level of IL-8 was correlated with the HMGB1 expression after propofol intervention (*p* < 0.05), showing a synchronous decrease; **c** The secretion of TNF-α and HMGB1 expression also showed the same trend of decrease, indicating a linear correlation (*p* < 0.05). LPS, lipopolysaccharide; IL-6, interleukin- 6; IL-8, interleukin-8; TNF-α, tumor necrosis factor-α; HMGB1, high-mobility group box 1; ^★^The relevant data were scaled to ensure that the trend of data changes

To determine whether the levels of IL-6, IL-8 and TNF-α are associated with HMGB1 expression, we performed the following correlation analysis. We found that the both between the level of IL-6 and HMGB1 expression in RAW 264.7 cells had a close linear correlation (correlation coefficient = 0.955; *p* = 0.046), either with LPS stimulation or by addition of propofol (Fig. 3a). For the expression level of IL-8, we also found that it had the same trend as HMGB1 expression, showing a linear correlation (correlation coefficient = 0.985; *p* = 0.015) (Fig. 3b). Moreover, the level of TNF-α and HMGB1 expression also showed the same trend, and the linear correlation displayed that the correlation coefficient was 0.996 and *p* value was 0.004 (Fig. 3c). From a statistical point of view, the correlation between TNF-α and HMGB1 (*p* = 0.004) was greater than that of IL-8 (*p* = 0.015) and IL-6 (*p* = 0.046).

## Discussion

Macrophages are considered to constitute an important component of immune defense. It is generally believed that when the body is inflamed, macrophages are activated and release a variety of cytokines and inflammatory mediators involved in the inflammatory response process. Lipopolysaccharide (LPS) has been recognized as one of the most potent inducers of inflammatory response [11]. Murine macrophage RAW 264.7 cells are an example of inflammatory cells [12]. Studies have shown that it can produce the corresponding cytokines and inflammatory mediators, such as TNF-α, IL-1β and IL-6 and so on as long as the mouse macrophage RAW264.7 cells are stimulated by LPS [13]. Propofol is widely used in the induction and maintenance of general anesthesia, and recent studies have shown that propofol can down-regulate the expression levels of HMGB1 messenger ribonucleic acid and protein [10]. Our study found that the expression of HMGB1 in RAW 264.7 cells in LPS intervention group was significantly increased, as compared with the blank control group after RAW 264.7 cells were stimulated by LPS for 16 h. Furthermore, compared with the blank control group, the expressions of HMGB1 in low-dose group of propofol and the high-dose group were also up-regulated. The results suggested that LPS stimulation increased the expression of HMGB1. Previous studies have found HMGB1 as an important inflammatory mediator and proinflammatory cytokine, which links in response networks of sepsis proinflammatory cytokine [10, 14–17]. LPS and various cytokines can stimulate the release of HMGB1, while HMGB1 can stimulates the synthesis of pro-inflammatory cytokines [8]. This proinflammatory functions and features of HMGB1 seem to be more useful in potential clinical application value than some of early inflammatory mediators. A series of basic studies have shown that HMGB1 is closely related to the inflammatory response, which mediates the release of multiple inflammatory mediators and proinflammatory factors and is therefore considered to be a potential therapeutic target in the sepsis study model [7, 15–17].

Since propofol is a short-acting intravenous anesthetic, it has the advantage of rapid induction and rapid resuscitation and is often used in a variety of animal experiments and surgical procedures [18]. As a new intravenous anesthetic, propofol may play a protective role in the body through the influence to cytokines produced by endotoxemia [10]. In our study, we analyzed whether the addition of propofol affect LPS-stimulated HMGB1 expression. We found that the expression of HMGB1 in propofol low dose group and high dose group were all significantly reduced and showed a concentration-dependent pattern, compared with the LPS intervention group. The results showed that propofol down-regulated the expression of LPS-stimulated HMGB1. A large number of laboratory studies have shown that as an oxygen free radical scavenger, propofol can inhibit lipid peroxidation, regulate antioxidant enzyme system, increase the antioxidant capacity of tissues and cells [1, 5, 15, 19]. Therapeutic concentrations of propofol also reduces nitrogen-containing compounds in mice induced monocyte-macrophage apoptosis and death [2]. Our study illustrated that propofol can effectively inhibit LPS-stimulated HMGB1 expression in RAW 264.7 cells, which indicating that propofol has an efficacy of anti-inflammatory. Studies have shown that HMGB1 can induce the synthesis and the secretion of inflammatory mediators in monocytes/macrophages, neutrophils and dendritic cells, these mediators can also strengthen the effect of HMGB1 secretion, forming a complex regulating secretion network of cytokine [7, 8, 16, 20, 21].

We also found that LPS stimulation increased the release of IL-6, IL-8 and TNF-α in RAW 264.7 cells. The study found that IL-6 has the effect of promoting inflammation, which is closely related to arthritis, tumor and obesity-related diseases. In addition, IL-8 is also involved in inflammatory response and plays an important role in tissue degradation [22]. Study shows that LPS is the main component of the outer wall of Gram-negative bacteria, which activates the nuclear transcription factor NF-κB through TLR-4 on the surface of macrophages to induce the release of a large number of inflammatory mediators and cells factors, which includes IL-6, IL-8 and TNF-α [23]. Our study showed an interesting phenomenon that the addition of propofol affects the release of LPS-stimulated IL-6, IL-8 and TNF-α. We found that either in propofol low-dose group or high-dose group, the levels of IL-6, IL-8 and TNF-α all significantly down-regulated, as compared with LPS intervention group (without propofol). The results suggested that propofol could effectively suppress LPS stimulated-RAW 264.7 cells to release IL-6, IL-8 and TNF-α, which indicated that it may play an important role of anti-inflammatory response. It has been suggested that in vitro propofol can block LPS-stimulated PBMC cells to produce IL-6 and IL-10 [24]. In addition to anesthetic effects, propofol seems to reduce the production and release of TNF-α [24]. In general, LPS can induce RAW 264.7 cells to IL-6, IL-8 and TNF-α, but propofol can inhibit the effect, which indicates that propofol may play a protective role in the development and progression of inflammation.

To determine whether the release of IL-6, IL-8 and TNF-α correlated with the HMGB1 expression in the course of LPS and propofol intervention, we performed a series of correlation analysis. We found that the expression of HMGB1 and the releases of IL-6, IL-8 and TNF-α in RAW 264.7 cells displayed a close linear correlation either with LPS stimulation or by addition of propofol. Studies show that adding HMGB1 in cultured human peripheral blood mononuclear cells can promote the expressions of TNF-α, IL-1, IL-6, IL-8 [7, 15, 16]. Recent experiment has shown that HMGB1 is an important inflammatory mediator in the regulation of hepatic ischemia-reperfusion injury and plays an important role in the early stages of tissue injury [17]. In addition, propofol can inhibit the production and release of HMGB1 in mouse macrophages stimulated by LPS via downregulating the expression of HMGB1 mRNA, and it also inhibit the activity of NF-κB [10]. Our results suggest that LPS can lead to the release of IL-6, IL-8 and TNF-α and thus may promote the inflammatory process. However, propofol has a significant inhibitory effect on IL-6, IL-8 and TNF-α in LPS stimulated RAW 264.7 cells. And propofol also significantly inhibited the expression of HMGB1 in LPS stimulated RAW 264.7 cells. More importantly, the release of IL-6, IL-8 and TNF-α intimately correlates with the expression of HMGB1. The results suggest that in addition to anesthetic effects, propofol may prevent systemic inflammatory responses, inhibiting the infection or non-infectious factors through reducing the release of some cytokines and inflammatory mediators, which may have a protective effect on the body’s emergency status.

## Conclusion

The expression of HMGB1 and the levels of IL-6, IL-8 and TNF-α were up-regulated in LPS-stimulated RAW 264.7 cells and supernatants. However, propofol down-regulated the expression of LPS-stimulated HMGB1 and reduced the LPS stimulated releases of IL-6, IL-8 and TNF-α. In addition, the levels of IL-6, IL-8 and TNF-α intimately correlated with the expression of HMGB1.

## Abbreviations

BSA: Bovine serum albumin
DNA: deoxyribonucleic acid
EDTA: Ethylene diamine tetraacetic acid
ELISA: Enzyme linked immunosorbent assay
FBS: Fetal bovine serum
HMGB1: High mobility group box-1 protein
ICAM-1: Intercellular cell adhesion nmolecule-1
IL-6: Interleukin- 6
IL-8: Interleukin- 8
LPS: Lipopolysaccharides
M ± SD: Mean ± standard deviation
NF-κB: Nuclear factor κB
NO: Nitric oxide
p38: Mitogen-activated protein kinase
PBS: phosphate buffer saline
PVDF: Polyvinylidene difluoride membranes
RAW 264.7: Mouse monocyte-macrophage leukemia cells
SDS-PAGE: Sodium dodecyl sulfate polyacrylamide gel electrophoresis
SPSS: Statistical product and service solutions
TBST: Mixture of tris-buffered saline and polysorbate 20
TLR-4: Toll- like receptor-4
TNF-α: Tumor necrosis factor-α
VCAM-1: Vascular cell adhesion molecule-1
